# The Diversity and Abundance of Small Arthropods in Onion, *Allium cepa*, Seed Crops, and their Potential Role in Pollination

**DOI:** 10.1673/031.011.9801

**Published:** 2011-07-29

**Authors:** M. K. Walker, B. G. Howlett, A. R. Wallace, J. A. Mccallum, D. A. J. Teulon

**Affiliations:** ^1^ New Zealand Institute for Plant and Food Research Limited, Private Bag 4704, Christchurch, New Zealand

**Keywords:** Diptera, exclusion cage, pollen flow, Thysanoptera, window trap

## Abstract

Onion, *Allium cepa* L. (Asparagales: Amaryllidaceae), crop fields grown for seed production require arthropod pollination for adequate seed yield. Although many arthropod species visit *A. cepa* flowers, for most there is little information on their role as pollinators. Small flower visiting arthropods (body width < 3 mm) in particular are rarely assessed. A survey of eight flowering commercial *A. cepa* seed fields in the North and South Islands of New Zealand using window traps revealed that small arthropods were highly abundant among all except one field. Insects belonging to the orders Diptera and Thysanoptera were the most abundant and Hymenoptera, Collembola, Psocoptera, Hemiptera, and Coleoptera were also present. To test whether small arthropods might contribute to pollination, seed sets from umbels caged within 3 mm diameter mesh cages were compared with similarly caged, hand-pollinated umbels and uncaged umbels. Caged umbels that were not hand-pollinated set significantly fewer seeds (average eight seeds/umbel, *n* = 10) than caged hand-pollinated umbels (average 146 seeds/umbel) and uncaged umbels (average 481 seeds/umbel). Moreover, sticky traps placed on umbels within cages captured similar numbers of small arthropods as sticky traps placed on uncaged umbels, suggesting cages did not inhibit the movement of small arthropods to umbels. Therefore, despite the high abundance of small arthropods within fields, evidence to support their role as significant pollinators of commercial *A. cepa* seed crops was not found.

## Introduction

Many crops are completely or partly dependent on arthropods for pollination (Free 1993; Cunningham et al. 2002), and in most cases, large conspicuous arthropods (body width > 3 mm), such as Hymenoptera (e.g. Apidae) and Diptera (e.g. Syrphidae and Calliphoridae), are presumed to be their key pollinators (Free 1993). Large insects, particularly bees, are also considered key vectors for pollen transport between crop fields that can result in unwanted cross pollination (Osborne et al. 1999; Cresswell and Osborne 2004; Cresswell and Hoyle 2006; Cresswell 2010). However, small arthropods (< 3mm body width) that are often overlooked as potential pollinators, can be very abundant within many crops (Lewis 1973; Mound 2004), e.g. brassica and onion in New Zealand (Howlett et al. 2009a & b; Walker et al. 2009). They could also contribute to long distance pollen movement due to their propensity to be carried via wind currents (Lewis 1997; Pathak et al. 1999). Therefore, understanding the diversity, abundance, and the contribution that small arthropods make to crop pollination is necessary to determine their value as crop pollinators, and to evaluate their potential role in moving pollen between crop fields and related weeds that may lead to unwanted hybridization. This is particularly important for vegetable seed production where seed quantity and purity are key factors in determining crop value.

Onion, *Allium cepa* L. (Asparagales: Amaryllidaceae), is a seed crop that is dependent on insect pollination for large scale seed production. Florets are not self-fertile (Delaplane and Mayer 2000), and wind and gravity are considered to play minimal roles in pollination (Free 1993). *A. cepa* flowers are known to attract a diverse array of large arthropods (Free 1993 and references within), and of these Hymenoptera (particularly bees) and Diptera are usually the most abundant flower visitors (Bohart et al. 1970; Howlett et al. 2009b) and key pollinators (Bohart et al. 1970; Currah 1981; Kumar et al. 1985). Although small flower-visiting arthropods have been noted, including Thysanoptera (Carlson 1964) and Diptera (Bohart et al. 1970; Howlett et al. 2009b), the composition of the small arthropod fauna present within flowering *A. cepa* fields and their role in pollination remain poorly defined.

In New Zealand, commercial *A. cepa* seed crops are grown in both the North and South Islands. Understanding the abundance, distribution, and diversity of small arthropods within flowering *A. cepa* seed crops and evaluating their potential role as pollinators will assist in pin-pointing those arthropods responsible for pollination and pollen flow. Future research can then focus on those arthropods that contribute significantly to pollination. Therefore, in this study a window trap survey of flowering *A. cepa* fields was conducted to assess the relative diversity and abundance of small arthropods in flowering fields in the North and South Islands of New Zealand over a 4-year period. Then exclusion cage experiments were conducted in a single *A. cepa* seed field to compare seed set in umbels only accessible to small arthropods with umbels exposed to all pollinators.

## Materials and Methods

### Surveys using window traps

**Survey regions and field locations.** Arthropod surveys employing window traps were used to assess the abundance and diversity of crop visitor assemblages in commercial *A. cepa* seed fields in the key seed-growing regions of the North (Wairarapa) and South Islands (Marlborough, Canterbury, and Otago) of New Zealand. Eight seed fields were surveyed over four years, two fields from each of the four regions (Table 1). Differences between land usage for each region are described in Howlett et al. (2009b). Arthropod surveys were spaced temporally (i.e. in different years) and spatially (in fields separated by > 2 km) (Table 1). The commercial fields contained a range of cultivars grown for hybrid seed production (consisting of a male fertile and male sterile line), except for the Wairarapa fields that were open-pollinated (hermaphrodite). *A. cepa* seed crops in all regions of New Zealand predominantly flower between the last week of December through the first week of February each season, with the seed harvested in February-March. Honeybee hives were spaced evenly throughout all fields at a stocking rate of 6/ha. Field size was estimated by measuring field circumference, and climatic data (temperature range and rainfall) were obtained from meteorological stations within 10 km of each field (NIWA) (Table 1). Surveys were undertaken over a 4 day (continuous 96 ± 2 hour) period at peak flowering (Howlett et al. 2009b).

**Table 1.**
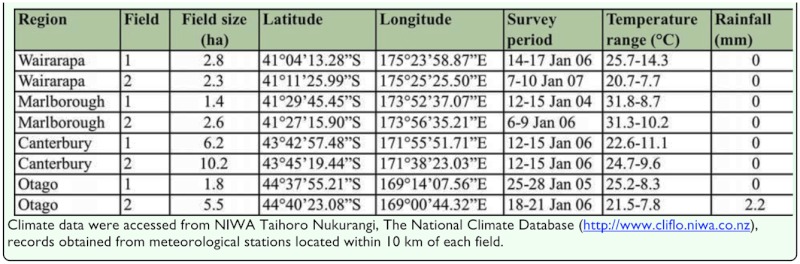
Onion crop field size, location, survey period, and climate data for the studied onion seed fields during the experimental period.

**Window traps and survey design** Window trap, survey design, arthropod identification, and storage methods were the same as described by Howlett et al. (2009b). In summary, window traps were used to collect arthropods from each corner (5 ± 1 m from the two field edges) and the centre of each *A. cepa* field. Trapped Diptera, the most common small arthropod group, were identified to family level. All other small and large arthropods were identified to order, with exception of Acari, which were identified to sub-class level. For each arthropod taxonomic group, the trap tallies from the five window traps per field were summed to give an overall total of arthropods trapped.

### Exclusion of large arthropods from onion inflorescences

Pollinator exclusion experiments were conducted to assess whether small arthropods contributed significantly to *A. cepa* pollination by using exclusion cages for large arthropods, and to test whether cages significantly inhibited small arthropod access to caged umbels. Experiments were conducted in a single field located at Barrhill (Canterbury Field 1 (Table 1)).

**Figure 1.**
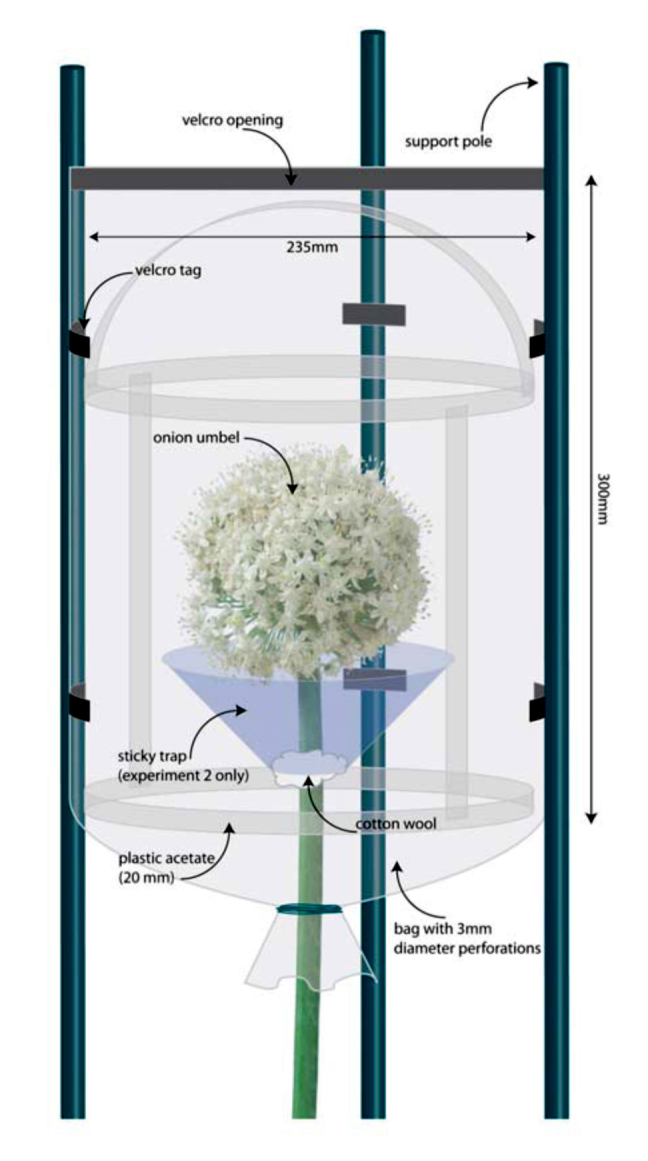
Cage designed to exclude large arthropods from onion umbels. Sticky traps to measure the type and abundance of arthropods were used in Experiment 2 only. High quality figures are available online.

Exclusion cages were designed to exclude large arthropods from individual umbels. Cages consisted of a clear acetate plastic (1 mm thick) cyclindrical support covered by a meshed bag (mesh hole size 3 mm in diameter) that contained a Velcro opening at the top (Figure 1). Three green plastic and metal supporting stakes (height 1.5 m, diameter 15 mm) were spaced equidistantly around the umbel. The cage structure was placed over the umbel and secured to each stake using Velcro tags sown to the bag (Figure 1). The bag was then tied around the umbel stem using string to close the cage.

**Experiment 1.** To assess whether small arthropods might contribute significantly to *A. cepa* pollination, a randomised complete block design with 10 replicates of three treatments laid out in two blocks (each containing five replicates) was used. The treatments were: (1) umbel enclosed in a cage, allowing the passage of small arthropods only, to assess seed set in the absence of large arthropods; (2) umbel enclosed in a cage, allowing the passage of small arthropods only and with hand cross-pollination (inflorescences were hand pollinated twice daily for a period of five days) to assess whether the cage design might inhibit seed set; and (3) uncaged umbels to assess seed set under open conditions in the presence of large and small arthropods. Umbels that had just begun flowering, (i.e. contained between one and 10 open flowers) were chosen as replicates, and open flowers were subsequently removed. Only male fertile line umbels were used to eliminate the complication of the male fertile versus male sterile effect on seed set in the hybrid seed crop. The first block of five replicates were spaced 5–8 m apart (the first replicate beginning 7 m inside the western field margin and the last replicate ending 42 m inside the field margin). The second block of five replicates was located near the eastern field margin and replicates were spaced similarly to block 1 (i.e. 7–42 m inside the field margin). Treatment umbels remained in the field until seed set (approximately 3 weeks). For each umbel the total number of fully developed seeds and aborted ovules was obtained. The percentage seed set was calculated from the total number of fully developed seeds per umbel and total number of ovules (seeds + aborted ovules). The angular transformed percentage seed set per umbel and the log-transformed [Log 10 (n + 1)] mean number of fully developed seeds per umbel were compared between treatments using Analysis of Variance (ANOVA).

**Table 2.**
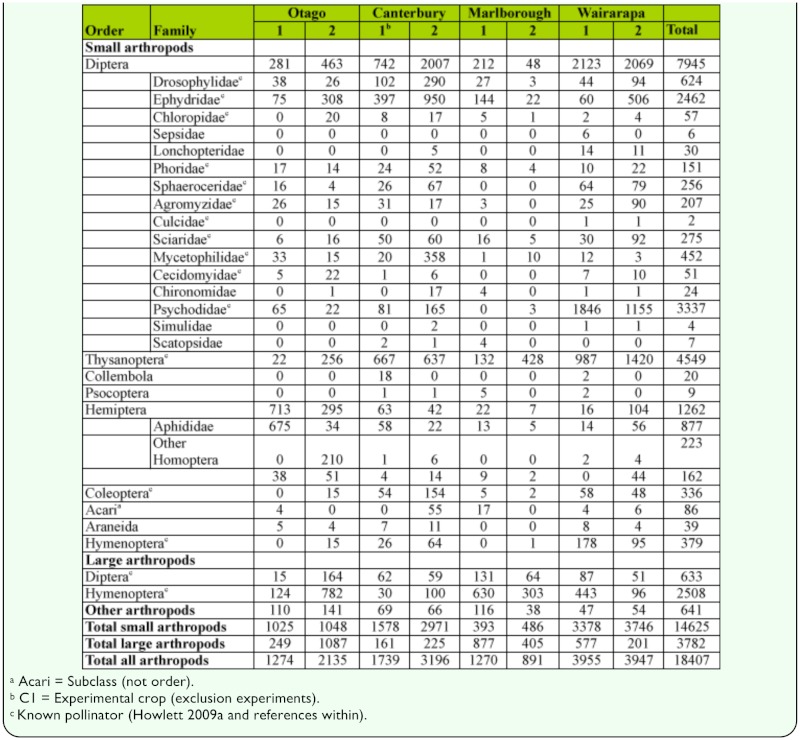
Total counts of small (< 3 mm body width) and large (> 3 mm body width) arthropod taxa collected from window traps from eight onion fields across four regions throughout New Zealand.

**Experiment 2.** To test whether the cages significantly inhibited small arthropod access to umbels, the type and abundance of small arthropod flower visitors inside and outside the cages was assessed using sticky traps. The traps consisted of a circular piece of clear acetate, with a central hole (diameter 15 mm). It was stapled around the umbel stem to form a funnel positioned within 15 mm of the base of the umbel with cotton wool placed between the stem wall and the acetate to protect the umbel stem from damage (Figure 1). A thin layer of Tangle-Trap (Insect Trap Coating: paste formula, The Tanglefoot® Company, www.tanglefoot.com) was then applied to the acetate to capture arthropods.

The experiment was conducted simultaneously with Experiment 1 and within the same field. Four 2 × 2 Latin squares were laid out in two blocks consisting of two treatments replicated eight times (four replicates per block). The treatments were: (1) caged umbels containing a sticky trap and (2) uncaged umbels containing a sticky trap, as a control. Replicates were spaced 5–8 m apart, (the first replicate beginning 5 m inside the field margin and the last replicate ending 35 m inside the field margin). The two experimental blocks were separated by 20 m from the Experiment 1 blocks. Traps were removed from the field after seed set (approximately 3 weeks). Arthropods adhering to the traps were sorted to order, or family level where possible, and counted. Because data consisted of counts with generally low numbers, a generalized linear model with Poisson error distribution and log link was used for analysis. Where replicate differences were small and their deviance less than the residual deviance (P > 0.4), the replicate deviance was pooled with the residual (Bancroft and Han 1983). If the residual deviance was less than the theoretical Poisson value of 1.0, the latter was used to test the difference between treatments. Percentage seed set per umbel and mean number of fully developed seeds per umbel were analyzed as for corresponding data from Experiment 1 and compared between corresponding treatments in that experiment (i.e. uncaged and caged umbels without sticky traps) using ANOVA. All statistical analyses were done using the GenStat statistical package (GenStat 2007). Arthropods within the experimental field were also sampled over a four-day period using window traps during the experimental period and the same survey design outlined above.

**Table 3.**
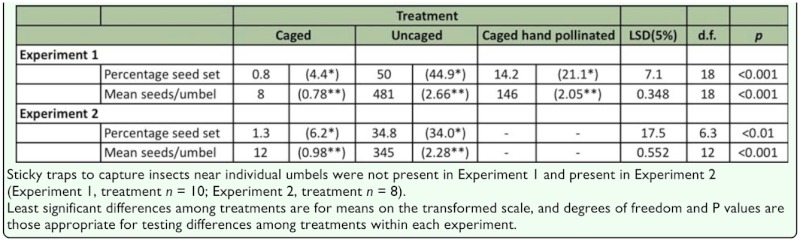
Raw mean, and in parentheses, means of *angular or **logio transformed data for percentage seed and total seed set per umbel, respectively.

## Results

### Window traps

The total number of arthropods counted in the window traps from all eight fields was 18,407. Small arthropods were substantially more abundant than large arthropods, representing 79.5% of total arthropods captured across the four regions of New Zealand (Table 2). Small arthropods were highly abundant in all eight fields across the four regions of New Zealand, ranging from 30.9% (Marlborough Field 1 (Table 1)) to 94.9% (Wairarapa Field 2 (Table 1)) of total arthropods captured per field, with 90.7% in the exclusion experiment field (Canterbury Field 1 (Table 1) (Table 2)).

Large arthropods were dominated by Hymenoptera (66.3% of total large arthropods) and Diptera (16.7%) (Table 2). Of the large Hymenoptera, *Apis mellifera* (L.) was the dominant species representing 77.5% of all individuals. For the Diptera, *Oxysarcodexia varia* (Walker) was the most abundant species representing 33.6% of all individuals. Small arthropods were dominated by Diptera (54.3% of total small arthropods) and Thysanoptera (31.1% of total small arthropods) (Table 2). There were 16 families of small-Diptera collected in the window traps (Table 2). The four most dominant families were Psychodidae (42% of total small-Diptera), Ephydridae (31%), Drosophilidae (7.9%), and Mycetophilidae (5.7%), all of which are known pollinators of plants or crop species (Howlett 2009b and references within).

### Exclusion experiments

**Experiment 1.** Percentage seed set per umbel varied significantly (P < 0.001) between all three treatments. It was lowest for the caged treatment at 0.8%, followed by the hand-pollinated treatment at 14.2%, and greatest in the uncaged treatment at 50.0% (Table 3). The mean number of fully developed seeds per umbel also varied significantly (P < 0.001) between all three treatments with caged being the lowest (8), followed by hand-pollinated (146), and uncaged (481) (Table 3).

**Table 4.**
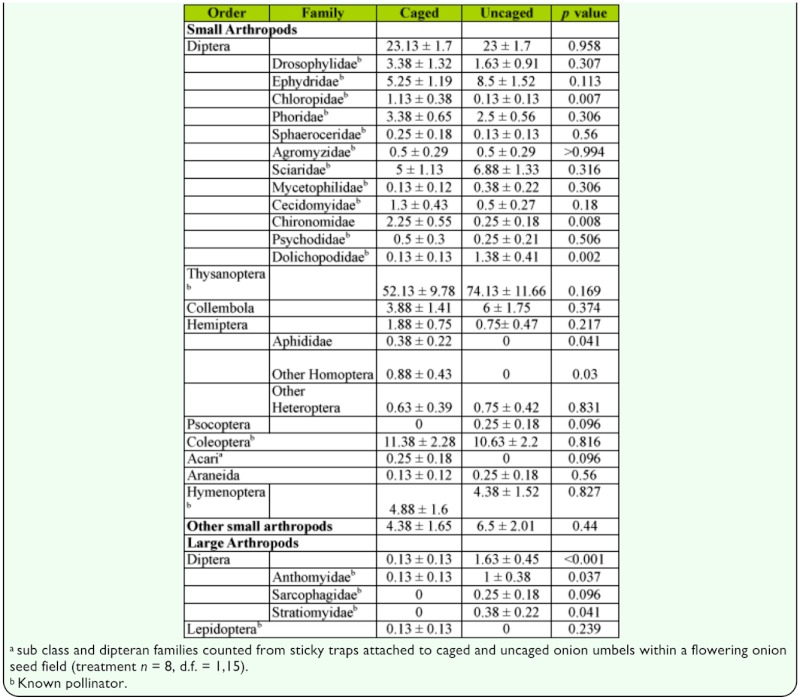
Mean ± SE number per trap of arthropod orders

**Experiment 2.** Like the first experiment, percentage seed set per umbel and mean number of fully developed seeds per umbel for the caged (with sticky traps) treatment were significantly less than those for the uncaged (with sticky traps) treatment (P < 0.01 and P < 0.001, respectively) (Table 3). Comparing Experiment 2 (sticky traps present) with Experiment 1 (no sticky traps present), there were no significant differences (P>0.05) in either percentage seed set per umbel or mean number of fully developed seeds between caged and uncaged treatments.

A diverse assemblage of small arthropods was collected on sticky traps in both caged and uncaged *A. cepa* umbels; with Diptera, Thysanoptera, Hymenoptera, and Coleoptera being the dominant orders (Table 4). The small arthropods collected from the sticky traps in the experimental field were similar to those collected in the window traps in the same field. Of the 11 small-dipteran families collected in window traps, 10 were also collected by sticky traps while the only order that was not represented in both window and sticky trap collections was Lepidoptera (collected only on sticky traps) (Table 4). There were no significant differences (P > 0.10) in sticky trap counts between caged and uncaged umbels for small-Diptera, small-Coleoptera, small-Hemiptera, small-Hymenoptera, Thysanoptera, and other small arthropods (Table 4). However, there were some significant differences between arthropod families within these groups with Chloropidae (P < 0.01), Chironomidae (P < 0.01), Aphididae (P < 0.05), and other Homoptera (P < 0.05) being significantly more abundant in caged umbels than in uncaged umbels, while Dolichopodidae (P < 0.01) were significantly more abundant in uncaged umbels, than in caged umbels (Table 4). Low numbers of large Diptera were also captured by sticky traps surrounding uncaged umbels which were not captured by traps surrounding caged umbels. The exception was a single Anthomyiidae collected by a trap on a caged umbel (Table 4). The specimen was estimated to be 3.5 mm in width and may have accidentally been trapped inside the cage as it was placed around the umbel.

## Discussion

### Abundance of small arthropods

Small arthropods (body width < 3 mm) were found to be very abundant in flowering *A. cepa* fields grown for commercial seed production throughout New Zealand. Window traps placed within peak flowering *A. cepa* fields captured more small arthropod individuals than large arthropod individuals in seven of eight fields. Moreover, the small arthropod individuals were found to represent at least nine different orders. For smallDiptera alone, 16 different families were represented in trap catches. Although small arthropods have previously been noted within flowering *A. cepa* fields, in most cases few details are provided on their identity, abundance, or diversity. Bohart et al. (1970) referred to the presence of tiny flies on flowering *A. cepa* umbels, however, they did not define their size or provide data on their abundance or diversity. Carlson (1964) noted that the presence of Thysanoptera on *A. cepa* umbels may have contributed to pollination, while Howlett et al. (2009b) noted the presence of small arthropods from several orders as being present within flowering *A. cepa* fields in New Zealand.

Small arthropod taxa sampled by window traps were similar between fields irrespective of location or sampling time, however, the relative abundances of the different taxa between fields varied by up to a factor of 10. This could have reflected regional differences, such as land use between field locations. Variation in land use and landscape features (e.g. hedgerows) are known to influence arthropod abundance (Tscharntke et al. 2005; Pollard and Holland 2006), and in this study land use was variable across the regions studied (Kirkpatrick 2005). In summary, horticultural industries, such as viticulture and orchards, were the major land users in the Marlborough region, while in the Canterbury region land was mainly used for intensive pastoralism and cash crops. In the Central Otago region, some land is used for intensive pastoralism and cash crops, but larger tracts of land are utilized for semi-intensive and extensive pastoralism (sheep and beef).

The type of small arthropods sampled in this study were also very similar to those sampled within flowering pak choi (*Brassica rapa* var. *chinensis*) fields throughout New Zealand (Howlett et al. 2009a; Walker et al. 2009). Of those small arthropod orders and dipteran families identified within pak choi fields by Howlett et al. (2009a), all were collected within the *A. cepa* fields in the present study. Of the Diptera, Scatopsidae was the only family present in *A. cepa* fields (at counts ≤ 4 per field) and absent in pak choi fields. Moreover, the study by Howlett et al. (2009a) recorded Ephydridae and Drosophilidae as the abundant dipteran families in most fields, similar to the finding in the present study for *A. cepa* fields. Thus, many of the common small arthropods present within *A. cepa* fields do not appear to be solely associated with flowering *A. cepa*. In New Zealand agroecosystems, many crops are spaced several kilometres apart and flower for periods of less than a month. For small insects that may be transported via wind over distances of several kilometres, an ability to utilize a variety of floral resources should increase the chance of finding food and shelter in these environments. Therefore, the similarity of small arthropods between crop species may reflect their ability to utilize many flowering plants. Moreover, issues regarding the role of small arthropods as vectors for pollen flow or as crop pollinators may be similar across a number of crop species. These may include arthropod movement within and between crops and their capability of carrying pollen.

This study used window traps to sample small arthropods. Window traps have been proven effective at sampling a wide range of arthropods within flowering crop fields, including small arthropods (Howlett et al. 2009b). Moreover, Howlett et al.'s (2009b) study across multiple peak flowering *A. cepa* and pak choi (*Brassica rapa* var *chinensis*) fields throughout New Zealand revealed strong correlations between the number of individuals observed on flowers and captured within window traps for a range of dipteran families and bee genera. It is possible that the relative abundance of different arthropod taxa captured in the trap samples may be under or over represented due to varying efficiency of traps towards capturing different taxa, however, for small Diptera and Hemiptera, high numbers observed within flowering of pak choi and *A. cepa* fields corresponded with high numbers captured in window traps across the same fields (Howlett 2009b).

### Small arthropods as potential onion pollinators

Caging umbels in mesh cages to exclude large arthropods of body width > 3 mm greatly reduced the amount of seed set within umbels (by approx. 60 and 30 times as measured by the two cage exclusion experiments, respectively). Moreover, hand-pollinated caged umbels still had 18 times the seed set of caged umbels with no hand pollination, suggesting small arthropods were not very effective pollinators of caged umbels. If the seed set recorded from caged umbels was solely due to small arthropod pollination, then small arthropods would need to be many times more abundant in these fields to cause significant seed set. Most other studies using exclusion or inclusion cages do not identify small arthropods as significant pollinators of *A. cepa*. Carlson (1964) recorded slightly higher levels of seed set from caged *A. cepa* umbels containing thrips compared with cages where all arthropods were excluded, but the difference was not significant. Woyke (1982) found that *A. cepa* within exclusion cages did not set any seed, however, Kumar et al. (1985) found that umbels within exclusion cages still set about a third of the seed that uncaged *A. cepa* set. These previous studies did not provide detail on the diversity and abundance of small arthropods that may have been present. In contrast, this study has demonstrated that diverse small arthropods are abundant within flowering *A. cepa* seed fields throughout New Zealand and that they are found in close proximity to *A. cepa* umbels.

The cages and/or the hand pollination technique used appeared to influence seed set, as hand-pollinated caged umbels had approximately one-third the seeds of uncaged umbels. Although, the cages did not affect the ability of the small arthropods to access these umbels. Most small arthropods were abundant around umbels regardless of whether large arthropods were excluded. Seed set may possibly have been influenced by other factors, particularly the effect of the cage on small arthropod behaviour (rather than abundance), but this was not measured. Other modes of pollination, such as wind and gravity, are considered possible but negligible for *A. cepa* (Free 1993).

This study did not find evidence that small arthropods significantly contributed to the pollination of a commercial *A. cepa* field despite being very abundant within the field. However, given the possibility of a cage effect (suggested by the reduced seed set in caged, hand-pollinated umbels compared to that in uncaged umbels), seed set from caged umbels with no hand pollination may have also been reduced. Therefore, small arthropods might play a greater role in the pollination of *A. cepa* than suggested by the findings of this study. It is also possible that hand pollination was not as effective as open pollination because it was done for only 5 days during the flowering period (however, more than 80% of flowers were estimated to be open during this time), or because of other technical difficulties not related to the cages, in which case the difference between these two treatments may not be due to a cage effect. In either case, the large difference in mean numbers of seeds set (18 fold) between the two caged treatments (those pollinated by hand versus those that were not) suggests that seed set from umbels exposed only to small arthropods is greatly reduced, irrespective of any cage effect that may have occurred.

The apparent abundance, diversity, and widespread occurrence of small arthropods in *A. cepa* and in other crops, such as *Brassica rapa* (Howlett 2009a), highlights the need to better understand their role as crop pollinators. To date, the role of small arthropods as crop pollinators has been documented in just a few crops (e.g. pollination of atemoya orchard crops by nitidulid beetles (Blanche et al. 2006) and cacao pollination by *Forcipomyia* spp. midges (Glendinning 1971; Soria et al. 1976; Soria et al. 1980)). However, they may be significant pollinators for many other crops. Likewise, they have the potential to contribute to pollen flow leading to crop contamination and hybridization between crop plants and related weeds. Future studies that assess the pollination efficiency of the most abundant and widespread small arthropods present within flowering crops would provide an important step for quantifying their contribution to crop pollination.

## References

[bibr01] Bancroft TA, Han C (1983). A note on pooling variances.. *Journal of the American Statistical Association*.

[bibr02] Blanche KR, Ludwig JA, Cunningham SA (2006). Proximity to rainforest enhances pollination and fruit set in orchards.. *Journal of Applied Ecology*.

[bibr03] Bohart GE, Nye WP, Hawthorn LR (1970). *Onion pollination as affected by different levels of pollinator activity*..

[bibr04] Carlson EC (1964). Effect of flower thrips on onion seed plants and a study of their control.. *Journal of Economic Entomology*.

[bibr05] Cresswell JE (2010). A mechanistic model of pollinator-mediated gene flow in agricultural safflower.. *Basic and Applied Ecology*.

[bibr06] Creswell JE, Hoyle M (2006). A mathematical method for estimating patterns of flower-to-flower gene dispersal from a simple field experiment.. *Functional Ecology*.

[bibr07] Cresswell JE, Osborne JL (2004). The effect of patch size and separation on bumblebee foraging in oilseed rape: Implications for gene flow.. *Journal of Applied Ecology*.

[bibr08] Cunningham SA, FitzGibbon F, Heard TA (2002). The future of pollinators for Australian agriculture.. *Australian Journal of Agricultural Research*.

[bibr09] Currah L (1981). Onion flowering and seed production.. *Scientific Horticulture*.

[bibr10] Free JB (1993). *Insect pollination of crops*.

[bibr11] Delaplane KS, Mayer DF (2000). *Crop Pollination by Bees*..

[bibr12] GenStat (2007). GenStat for Windows, 10^th^ ed. VSN International..

[bibr13] Glendinning DR (1971). Natural pollination of cocoa.. *New Phytologist*.

[bibr14] Howlett BG, Walker MK, McCallum JA, Teulon DAJ (2009a). Small flower-visiting arthropods in New Zealand pak choi fields.. *New Zealand Plant Protection*.

[bibr15] Howlett BG, Walker MK, Newstrom-Lloyd LE, Donovan BJ, Teulon DAJ (2009b). Window traps and direct observations record similar arthropod flower visitor assemblages in two mass flowering crops.. *Journal of Applied Entomology*.

[bibr16] Kirkpatrick R (2005). *Bateman contemporary atlas New Zealand: The shapes of our nation*.

[bibr17] Kumar J, Mishra RC, Gupta JK (1985). The effect of mode of pollination on allium species with observation on insects as pollinators.. *Journal of Apicultural Research*.

[bibr18] Lewis T, Lewis T (1997). Flight and dispersal.. *Thrips as crop pests*.

[bibr19] Lewis T. (1973). *Thrips: Their biology, ecology and economic importance*..

[bibr20] Ministry of Agriculture and Forestry. (2008). *Horticulture and arable monitoring report 2008 Maf policy*..

[bibr21] Mound LA (2004). Australian Thysanoptera Biological diversity and a diversity of studies.. *Australian Journal of Entomology*.

[bibr22] NIWA Taihoro Nukurangi. The National Climate Database..

[bibr23] Osborne JL, Clark SJ, Morris RJ, Williams IH, Riley JR, Smith AD, Reynolds DR, Edwards AS (1999). A landscape-scale study of bumble bee foraging range and constancy, using harmonic radar.. *Journal of Applied Ecology*.

[bibr24] Pathak SC, Kulshrestha V, Choubey AK, Parulekar AH (1999). Insect drift over the northern Arabian sea in early summer.. *Journal of Biosciences*.

[bibr25] Pollard KA, Holland JM (2006). Arthropods within the woody element of hedgerows and their distribution pattern.. *Agricultural and Forest Entomology*.

[bibr26] Soria SdJ, Wirth WW, Chapman RK (1980). Insect pollination of Cacao in Costa Rica. 1. Preliminary list of the ceratopogonid midges collected from flowers.. *Revista Theobroma*.

[bibr27] Soria SdJ, Wirth WW, Flores F JD (1976). Identity of the midges *Forcipomyia* spp. (Diptera, Ceratopogonidae) associated with the pollination of Cacao in Ecuador (preliminary note).. *Revista Theobroma*.

[bibr28] Tscharntke T, Klein AM, Kruess A, Steffan-Dewenter I, Thies C (2005). Landscape perspectives on agricultural intensification and biodiversity - ecosystem service management.. *Ecology Letters*.

[bibr29] Walker MK, Howlett BG, McCallum JA, Wallace AR, Teulon DAJ (2009). Small Arthropods as pollinators in a New Zealand pak choi field trial.. *New Zealand Plant Protection*.

[bibr30] Woyke H (1982). Research on onion pollination.. *Zapylanie roslin warzywnych*. III Seminarium, 28 II - 1 III 1979..

